# Screening Protocol for Early Identification of Brazilian Children at Risk for Dyslexia

**DOI:** 10.3389/fpsyg.2017.01763

**Published:** 2017-10-27

**Authors:** Giseli D. Germano, Alexandra B. P. de C. César, Simone A. Capellini

**Affiliations:** ^1^Investigation Learning Disabilities Laboratory, Department of Speech and Hearing Sciences, São Paulo State University “Júlio de Mesquita Filho” (UNESP), Marília, Brazil; ^2^Department of Special Education, São Paulo State University “Júlio de Mesquita Filho” (UNESP), Marília, Brazil; ^3^Speech and Hearing Sciences Department, São Paulo State University “Júlio de Mesquita Filho” (UNESP), Marília, Brazil

**Keywords:** reading, dyslexia, early identification, phonological awareness, assessment

## Abstract

Early identification of students at risk of dyslexia has been an educational challenge in the past years. This research had two main goals. First, we aimed to develop a screening protocol for early identification of Brazilian children at risk for dyslexia; second, we aimed to identify the predictive variables of this protocol using Principal Component Analysis. The major step involved in developing this protocol was the selection of variables, which were chosen based on the literature review and linguistic criteria. The screening protocol was composed of seven cognitive-linguistic skills: Letter naming; Phonological Awareness (which comprises the following subtests: Rhyme production, Rhyme identification, Syllabic segmentation, Production of words from a given phoneme, Phonemic Synthesis, and Phonemic analysis); Phonological Working memory, Rapid naming Speed; Silent reading; Reading of words and non-words; and Auditory Comprehension of sentences from pictures. A total of 149 children, aged from 6 years to 6 and 11, of both genders who were enrolled in the 1st grade of elementary public schools were submitted to the screening protocol. Principal Component Analysis revealed four factors, accounting for 64.45% of the variance of the Protocol variables: first factor (“pre-reading”), second factor (“decoding”), third factor (“Reading”), and fourth factor “Auditory processing.” The factors found corroborate those reported in the National and International literature and have been described as early signs of dyslexia and reading problems.

## Introduction

Early identification of students at risk for dyslexia has been an educational challenge in the past years. Although scientific research has explored the nature, etiology, assessment, and intervention of this learning disorder, educators are still having a hard time recognizing its signs, which suggest that a child might be at risk for reading failure without being identified. Such early identification should allow interventions to be implemented before a downward spiral of underachievement, lowered self-esteem and poor motivation sets in (Shaywitz and Shaywitz, 2005; Kirby et al., 2010; Snowling, 2013; Hulme et al., 2015). In Brazil, this topic is still fairly new; research has been carried out since 2009 (Capellini et al., 2009, 2015; Andrade et al., 2011; Fadini and Capellini, 2011; Fukuda and Capellini, 2011, 2012), attempting to develop a screening protocol for early identification of children at risk for dyslexia. These studies have reported that phonological awareness, verbal working memory, and rapid naming correspond to the central phonological mechanisms of acquiring reading and writing that have been also reported by De Jong and Van der Leij (1999). However, none of them explored the predictive values of each variable and their impacts on the development of a minimal protocol for early identification. Hulme and Snowling (2014) have considered letter knowledge, phonological awareness, and rapid automatized naming as predictors which are important, since it makes it possible to differentiate individual performance in students at risk for dyslexia, regarding decoding skills in alphabetic languages in the early stages. Studies have reported that students with developmental dyslexia may present as manifestations difficulties with accurate or fluent word recognition and spelling, even when they had received adequate instruction, and have no signs of fails in intelligence and sensory abilities (Shaywitz and Shaywitz, 2005; Kirby et al., 2010; Snowling, 2013; Hulme et al., 2015). The authors also described that dyslexia is the result of several risk factors, and children who have language difficulties in the first school years are usually considered as being at high risk for learning disabilities. Another important issue about dyslexia refers about family history, which also plays an important role as a predictor of literacy outcome in the preschool years. However, assessment’s protocols will only help to identify the risk after the children start literacy at school, when they will have formal instruction about letter knowledge, phonological awareness, and rapid automatized naming (RAN); together these skills provide good sensitivity and specificity as a screening battery. Furthermore, the consensus between these studies is that the first signs of dyslexia include delays in speech and language development, with phonological memory (non-word repetition) and expressive language (naming) skills being particularly affected, as mentioned in the studies of Carroll et al. (2014) and Thompson et al. (2015).

The relationship between phonological awareness, rapid naming, and reading in alphabetic languages has been documented in the literature over the last decades. Germano et al. (2014) described that Brazilian Portuguese language has an alphabetic system and that most words can be successfully read through phonological decoding, according to grapheme–phoneme correspondences (Pinheiro et al., 2008). Scliar-Cabral (2003) described that reading in Brazilian Portuguese is considerate to be transparent since it presents a set of one-to-one graph phonological relations, that is, univocal relations, and a set of inconsistent relations, many of which are governed by rules. Thus, a characteristic of reading processing in Brazilian Portuguese is that it can be performed, almost successfully, only when the grapheme-phoneme matching rules are known and phonological decoding is used, mostly at the beginning of literacy acquisition. With regard to writing, spelling of Brazilian Portuguese is considered as being more opaque. The reason is because, in general, writing is considered a more complex cognitive process that requires intention, selection, planning, monitoring, and revision, as well as a specific coding process (Godoy and Pinheiro, 2013). Therefore, learning to read and write implies a deliberate reflection of speech, promoting metalinguistic awareness (Bradley and Bryant, 1983; Hayes and Slater, 2008; Manz et al., 2010). Learning to read requires highly complex task, such as visual integration, orthographic, phonological, and semantic information. For example, in the dual route model of reading aloud (Coltheart et al., 2001; Ziegler et al., 2008), the reading process also requires a series of interacting stages, from letter feature detection to phonological output processes. This process was divided into two major routes: the lexical orthographic route and the non-lexical phonological route. The lexical route is important because allows the correct pronunciation of irregular words, while the non-lexical route allows the pronunciation of novel words and non-words, using not only phonological processes but also letter perception. The reading circuit is composed of neural systems including phonology, morphology, syntax, and semantics, as well as other processes such as visual and orthographic processes, that requires memory, attention, comprehension, eye movements, and cognition. By having these skills, the reader develops the so-called automatism, reading with adequate precision and speed. When this process becomes automatic, the effort toward the act of reading becomes less apparent (Norton and Wolf, 2012). Despite the vast international literature on this theme, there is lack of research on these precursors in Brazilian Portuguese Language, concerning first graders. In Brazilian Portuguese language, most words can be successfully read using phonological decoding, and even reading fluency reflect the ability of the reader to use grapheme-phoneme correspondences (Pinheiro et al., 2008). In Brazilian Portuguese, beginner readers, from first to third graders predominates the use of phonological route, but with age, performance gradually relies lexical route, such as knowledge and sight word vocabulary (Oliveira and Capellini, 2010; Mota et al., 2012). Phonological awareness is one of the most important precursor skill of reading and spelling and also one important predictors of the word recognition difficulties that characterize developmental dyslexia, one of the most common learning disorders, as reported in Peterson and Pennington (2012) and Skeide et al. (2015). Phonological awareness is the ability to identify, distinguish, and manipulate sounds within spoken language, and its importance to reading is widely acknowledged; thus, children who are able to identify and manipulate individual sounds have good academic performance. Impairment to the availability of early phonological skills can hinder subsequent reading progress (Duncan et al., 2013).

Studies have reported that the development of phonological awareness skills occurs in a sequential pattern, beginning in the 1st months of a child’s life, before entering school. However, these skills have been described to have an important role in reading acquisition, as the perception that speech has an underlying phonemic structure allows storage in long-term phonological memory, using the generative mechanism of phonological memory, which converts spellings into phonology (Chard and Dickson, 1999; Cervera-Mérida and Ygual-fernández, 2003; Gombert, 2003; Hayes and Slater, 2008; Germano and Capellini, 2011). Along with phonological awareness, phonological memory or verbal short-term memory (capacity of temporary storage based on sound information) has been highlighted as a component of phonological processing, that is required in reading development (Wagner and Torgesen, 1987; Gathercole et al., 1999; Alloway et al., 2005). Thus, phonological memory has an important role for vocabulary acquisition, because it provides a temporary phonological representation of unfamiliar words, and later it will be responsible for an enduring representation in long-term memory (Gathercole and Baddeley, 1989; De Jong and Olson, 2004). It also contributes to the acquisition of letter knowledge (De Jong and Olson, 2004), facilitating the word identification when grapheme–phoneme correspondence rules is necessary, and facilitates text comprehension because allows children to recuperate words they have already read.

Hulme et al. (2015) have reported that the development of reading skills requires underlying ability of oral language abilities. Phonological skills has a causal influence on the later development of early word-level literacy skills, which has an impact in reading-comprehension, involving (semantic and syntactic) language skills. The authors presented a longitudinal study comparing children at familial risk for dyslexia, children with preschool language difficulties, and typically developing control children. Theirs findings described that as preschool measures of oral language it was found that phoneme awareness and grapheme-phoneme knowledge were important to acquire before school entry, which in turn predicted word-level literacy skills shortly after school entry. These results were indicated also for both typically developing children and those at risk of literacy difficulties. The authors highlighted the importance of oral language skills for the development of both word-level literacy and reading comprehension.

In addition, less speed in naming may reflect difficulty in the integration of cognitive and linguistic processes involved in fluent reading (Araújo et al., 2016). Studies (Jones et al., 2010; Araújo et al., 2016) using the Rapid Automatized Naming Test (RAN) (Denckla and Rudel, 1976), which was designed to measure the speed at which a series of highly familiar items such as letters, digits, objects, and colors can be named. As a cognitive requirement, visual naming represents a demanding array of attentional, perceptual, conceptual, memory, lexical, and articulatory processes. Wolf et al. (2000) argued that this, in turn, RAN has played an important rule for identification or recognition processes, which integrate information of present stimulus with known mental representations, quality that will influence the speed of processing. Lexical processes, that include semantic, phonological access and retrieval processes, can be integrated with cumulative information. After the cognitive processes, motor commands translate this phonological information into an articulated name. The entire process occurs within 500 ms. Difficulties have been found to be invariant across languages (Brizzolara et al., 2006; Capellini and Conrado, 2009; Araújo et al., 2010). One of the reasons for using naming speed as part of reading evaluations is because naming speed and reading are similar. According to Kirby et al. (2010), in both RAN and oral reading subjects are solicited to move their eyes sequentially across the page, encode the stimulus that they are focusing on, access the mental representation of that stimulus, and then activate the associated motor commands that allows the subject to name that stimulus. Before the first motor commands is completed, the eyes must move on to the next stimulus, and so on. Just as in reading, the eyes must make a sweep back to the beginning of the next line. Several studies have justified the relationship between word reading and RAN concerning phonological deficits or phonological processing (Morris et al., 1998; Vaessen et al., 2009).

According to Thompson et al. (2015), identifying children with dyslexia or at risk for dyslexia means assessing the probability that a group of variables will identify positive cases of dyslexia (sensitivity), aiming to avoid false positives (specificity). Thus, the present study discusses the hypothesis that precursors of dyslexia described in Brazilian and international literature, such as knowledge of the alphabet, phonological awareness, working memory, rapid automatic naming, visual attention, reading words, and non-words, could be addressed for early identification of first grade children at risk for dyslexia in Brazil. This research had two main goals. First, we aimed to develop a screening protocol for early identification of children at risk for dyslexia; second, we aimed to identify the predictive variables of this protocol using Principal Component Analysis.

## Materials and Methods

In the first part of this study, the steps to develop this screening protocol, such as variable selection, will be described based on the literature. This screening protocol was developed to be used as a universal screening for first grade children and as part of the Tier 1 of the response to intervention (RTI) model. According to Johnston and Kirby (2006), Tier 1 aims to identify the risks for behavioral and learning problems using procedures based on the academic curriculum of these children; therefore, it would be possible to verify if these children reached the expected results at their grade level. Capellini et al. (2015) used the Screening Protocol for the Early Identification of Reading Problems in Brazilian children at risk for dyslexia as part of a RTI study. Of the 156 students that were evaluated by these authors using the protocol, 62 fulfilled the risk criteria (performance below the 25th percentile for at least 51% of the Protocol variables). The students were submitted to phonological intervention, and the results obtained in the post-tests indicated that 12 students continued to be at risk, according to their performance. These students underwent a multidisciplinary evaluation to confirm the diagnosis.

Because reading involves multiple linguistic, visual, and attentional processes, it is likely that variable patterns of weaknesses may contribute to reading difficulties among children, as mentioned by Norton and Wolf (2012). However, the present study considered the recent investigations that have demonstrated that dyslexic children may have difficulties in underlying processes (e.g., phonological awareness and rapid naming test) and difficulties with RAN, related to visual attention processing (Franceschini et al., 2012; Germano et al., 2014). Taking that into consideration, the development of the screening protocol for early identification of reading problems (Capellini et al., 2009, 2015, 2017) was based on a literature review to identify the skills for effective reading and writing. The Protocol was composed of seven cognitive-linguistic skills divided into seven tests. The tests and justification for their selection are shown in **Table 1**.

**Table 1:** List of variables and justification for the development of the screening protocol for early identification of children at risk for dyslexia (Capellini et al., 2017).

Tests of the screening protocol	Justification for test selection
(1) Letter-naming	Pennington and Lefly (2001) stated that Letter-name knowledge is at the intersection between spoken and written language because letters are the written representations of phonemes or combinations of phonemes. It is plausible that the ability to learn letter names depends on underlying phonological development. As pointed out by Share (1995), letter names are, after all, non-words and the ability to repeat and remember non-words.
(2) Phonological Awareness: composed by the subtests	As reported by Brunswick et al. (2012), whereas awareness of larger phonological units, such as the syllable and onset–rime, develop independently of reading instruction in 3-to 5-year-olds (Bradley and Bryant, 1983; Badian, 2001; Gipstein et al., 2001), awareness of smaller units of sound, such as the phoneme, usually develop later as a result of reading development (De Jong and van der Leij, 1999; Cardoso-Martins and Pennington, 2004; Ziegler and Goswami, 2006).
(2.1) Rhyme production	
(2.2) Rhyme identification	
(2.3) Syllabic segmentation	
(2.4) Production of words from a given phoneme	
(2.5) Phonemic synthesis	
(2.6) Phonemic analysis	
(3) Phonological working memory (repetition of words and non-words)	Articulatory loop (phonological working memory system) is thought to be responsible for the temporary storage of verbal information, while other cognitive tasks, such as verbal reasoning or auditory and reading comprehension, are performed (Baddeley, 1986). The task of repeating single non-words is particularly appropriate for use with young children. Throughout the course of childhood, the child, who seems to be innately equipped with both, desire and facility to learn new words, encounters many thousands of unfamiliar words. Exposure to unfamiliar phonological forms is a natural and common occurrence for the child. A further issue of interest is whether non-word repetition ability shares a developmental link with reading achievement. Previously reviewed findings indicate that such a relationship might well exist. Impaired non-word repetition skills have consistently been shown to be characteristic of poor readers and children classified as dyslexic, as described by Gathercole et al. (1994)
(4) Rapid naming speed	According with Jones et al. (2010), naming-speed on these tasks is proposed to assess low-level factors involved in reading fluency, such as: attention to the stimulus bi-hemispheric visual processes responsible for feature detection; matching of feature and pattern encoding to stored orthographic representations; integration of visual and phonological information; and motor activation leading to articulation. Non-alphanumeric stimuli are preferred for use with young students or those who may not have learned letters and digits well enough to be “highly familiar” with them (Wolf and Bowers, 1999; Jones et al., 2010; Kirby et al., 2010).
(5) Silent reading	Measures of silent reading typically include a decision component, such as semantic categorization, sentence verification, or lexical decision. In addition, the main goal in silent reading is to comprehend and assimilate the meaning of the text, which relies on the grapheme-to-semantic decoding in the lexical route (Galin et al., 1992; van den Boer et al., 2014; Zhao et al., 2016). This protocol was based on semantic categorization via lexical processing. Whereas phonological representations might be activated in both silent and oral reading, computation of a phonetic code is specific to oral reading (van den Boer et al., 2014).
(6) Reading words and non-words	Given the nature of the written language, in order to learn to read, the beginning reader needs to decode the written words into speech units and then comprehend the words (and sentences/discourse) to derive meaning, according to The Simple View of Reading (Gough and Tunmer, 1986; Hoover and Gough, 1990). According to this view, reading is composed of word recognition and language comprehension. Word recognition is the translation of print into language (i.e., sounds and words) and comprehension is the making sense of language (Rack et al., 1992; Catts and Hogan, 2003). Moreover, word recognition is a good predictor of reading comprehension performance in children at the beginning of reading acquisition (Chaves-Sousa et al., 2016).
(7) Auditory comprehension of sentences from picture.	Montgomery (1995) argued that sentence comprehension requires that previous information be stored temporarily while new, incoming information is processed. Clark and Clark (1977) have proposed that phonological memory is critical to comprehension because listeners presumably store entire sentences in a phonological input store until all syntactic and semantic analyses have been completed.


After selecting the tests, the next step concerned the choice of linguistic stimuli to compose the Protocol. This study was based on a phonological perspective called linear model and on the hierarchical model (Câmera, 1970a,b; Selkirk, 1982). The screening protocol was composed of words from a word bank created for this study; these words were extracted from 1th to 5th grade textbooks (elementary school) written in Portuguese (Germano and Capellini, 2008; Germano, 2011). This word bank included words belonging to different word classes or parts of speech, such as pronouns, prepositions, adjectives, adverbs, verbs, and nouns. Exclusion criteria were as follows: pronouns, prepositions, words that could vary according to the class or grammatical category, gender, and agreement, which happens when a word changes form depending on the other words to which it relates (e.g., adjectives, adverbs, verbs). In addition, as linguistic criteria (Brazilian Portuguese Language), words that had one of the following characteristics were excluded: (1) Syllable reduction (for example, the word “fósforo” (phosphorus) pronounced as [fósfuru]∼[fosfru]. (2) Open and close vowels (for example, the word “bolacha” (cookie) pronounced as [ô] and “bola” (ball) pronounced as [ó]). (3) Words with diphthong and hiatus [for example, the word “vaidade” (vainity) can be pronounced as “vai.da.de,” “va.i.da.de”] and/or monotongation [for example, the word “caixa” (box) can be pronounced as c[aj]xa, c[a]xa]. (4) Words with nasal vowels [for example, “orgão” (organ), “homem” (man)]. (5) Tonicity of syllables containing vowel sounds (word selection was made based on the stressed syllable position, and the stressed syllable was in the same position in the target word and in word in the correct answer. (6) Neutralization [e.g., the word “pepino” (cucumber) can be pronounced as “p[e]pino” or “p[i]pino”]. (7) Consonant vocalization (e.g., the pronunciation of words with a consonant corresponding to a post-vowel velar phoneme/l/ may change, and thus it can be pronounced as /u/ or /w/). Most of the words used in Brazilian Portuguese had simple syllable structure, such as consonant-vowel, consonant-vowel-consonant, consonant-consonant-vowel. The screening protocol developed was applied to first graders.

The second goal of this study was to identify the predictive variables of this protocol using Principal Component Analysis. This study was approved by the Research Ethics Committee of the University *Júlio de Mesquita Filho* (FFC/UNESP, São Paulo State University - School of Philosophy and Sciences), Protocol No. 0663/2013.

### Participants

A total of 149 children, aged from 6 years to 6 years and 11 months, of both genders, who were enrolled in the 1st grade of elementary public schools participated in this study. Parents and/or guardians of all the participants signed an informed consent form. Exclusion criteria for participation in the study were as follows: children with sensory, motor, or cognitive impairment and children whose parents/guardians did not sign the Informed Consent form; inclusion criteria: children whose parents/guardians signed the Informed Consent form and children without sensory, motor, or cognitive impairment, according to information in the school records. Two schools with similar socio-economic status and high rating level in the Secretaria da Educação do Estado de São Paulo (2014) (System of Evaluation of School Performance of the State of São Paulo) participated in this study.

### Procedures

All participants were submitted to the Screening Protocol for Early Identification of Reading Problems (Capellini et al., 2015). The protocol was applied individually in a 50-min session. The protocol was composed of seven cognitive-linguistic tests. Each test was composed of two training trials and test stimuli. The training trials were not scored. During the training trials, the children were informed that the Examiner could offer further explanation about what was being asked and that the Examiner could repeat the stimulus, if necessary. During the test, the Examiner explained that the stimulus could be repeated only once. The rating scale values for Punctuation were: “one” for a correct answer and “zero” for an incorrect answer or a blank. Children marked their answers on an Answer Sheet. The screening protocol was composed of the following tests:

(1) Letter-naming test. Letters of the alphabet were presented randomly to the children, and they were asked to name the letter shown. Children were presented with visual stimuli with 12 pt Arial uppercase letters. A total of 23 stimuli were shown.
(2) Test of phonological awareness. Stimuli were orally presented without visual cues. This test was composed of the following subtests:
  (2.1) Subtest of Rhyme Production. The Examiner presented a word, and the student was asked to say a word that ended with the same sound. This subtest comprised 20 words (target stimuli). Example: target word: “cola” (glue) and expected answer: “bola” (ball).
  (2.2) Subtest of Rhyme Identification. The Examiner presented a series of three words, and the student was asked to provide a pair of rhyming words (words that sound the same at the end). Twenty groups of three words were presented. Example: a series of three words, “milho” (corn), “baleia” (whale), and “filho” (son). Expected answer: “milho/ filho.”
  (2.3) Subtest of Syllabic Segmentation. Students were presented with twenty-one words one at a time. The words were selected according to number of syllables (from 2 to 4 syllables), and the children were asked to dived them into syllables. Example: target stimulus: “vaca” (cow). Expected answer: “va – ca.”
  (2.4) Subtest of Production of words from a given phoneme. A sound/phoneme of the alphabet was presented to the students, and they were asked to say a word beginning with the same sound. Example: target stimulus: phoneme /a/. Expected answer: “asa” (wing).
  (2.5) Subtest of Phonemic Synthesis. Twenty-one words were presented one at time to the students. The Examiner pronounced each phoneme of the word and asked the children to combine them forming a word. The words were selected according to the number of syllables (from 2 to 4 syllables). Example, target stimuli: /k/ /a/ /f/ ε/. Expected answer: “café”/ coffee.
  (2.6) Subtest of Phonemic analysis. Twenty-one words were presented one at time to the students. The Examiner pronounced the words and asked the children to divide them by pronouncing each phoneme/sound of each word. The words were selected according to the number of syllables (from 2 to 4 syllables). Example, target stimulus: “bola/ ball.” Expected answer: /b/ /ó/ /l/ /a/.
  (2.7) Subtest of Identification of the initial sound/phoneme. Twenty-one words were presented one at time to the students. The Examiner pronounced a word and asked the children to say the initial sound/phoneme of the word out loud. Example, target stimulus: “boca/mouth.” Expected answer: /b/.
(3) Subtest of Phonological Working memory. Twenty-four non-words were presented one at time to the students, and they were asked to repeat the words as they heard them. Repetition was allowed only once. The non-words varied in length (from 1 to 6 syllables).
(4) Test of Rapid Naming Speed using pictures. Children were presented with flashcards containing seven lines and five columns with colorful picture images: car, ball, duck, house, and key. First, the students were asked to identify each picture stimulus, aiming to verify their recognition. Afterward, they were instructed to say the name of each stimulus as quickly as possible. The examiner used a chronometer to record the time.
(5) Silent reading. Students were presented with 10 colored pictures, below which there were two words, and they were asked to identify the word that described the picture. The words in the picture belonged to different semantic categories.
(6) Reading of words and non-words. A list with 20 words and 20 non-words was presented to the students, and they were asked to read them aloud. Children were presented with visual stimuli with 12 pt Arial uppercase letters. A total of 23 stimuli were shown.
(7) Auditory Comprehension of sentences from pictures. Twenty incomplete sentences with illustrations or figures were presented to the students. The sentences with a missing part were orally presented by the examiner. The children should listen to the sentence and name the missing part, according to the picture.

## Results

Statistical analysis was carried out using the SPSS (Statistical Package for Social Sciences), version 23.0. Some descriptive statistics are shown in **Table 2**.

**Table 2:** Distribution of mean (*M*), standard deviation (*SD*), minimum (min), and maximum values (max), and students’ test scores at the 25th and 75th percentiles using the proposed protocol.

Tests	*N*	Minimum	Maximum	*M*6	*SD*	25%	Median	75%
LN (/23)	149	5.00	23.00	21.66	2.88	21.00	23.00	23.00
RP (20)	149	0.00	20.00	11.42	6.96	5.00	13.00	18.00
RI (/20)	149	0.00	20.00	15.45	5.09	12.50	18.00	19.00
SS (/21)	149	0.00	21.00	19.38	3.41	19.00	21.00	21.00
PWPh (/21)	149	0.00	22.00	18.68	3.53	18.00	20.00	21.00
PhS (/21)	149	0.00	21.00	2.76	5.66	0.00	0.00	1.00
PhA (/21)	149	0.00	20.00	2.72	4.84	0.00	0.00	4.00
IPh (/21)	149	0.00	21.00	14.23	8.25	5.00	20.00	21.00
WM (/24)	149	1.00	24.00	20.54	3.05	19.50	21.00	23.00
RAN (seconds)	149	20.00	80.00	43.44	10.63	35.00	42.00	51.00
SR (/10)	149	2.00	10.00	8.95	1.52	8.00	10.00	10.00
RWNW (/40)	149	0.00	40.00	17.15	16.08	0.00	16.00	34.00
AC (/20)	149	0.00	28.00	19.32	2.51	19.00	20.00	20.00


*LN, letter-naming; RP, rhyme production; RI, rhyme identification; SS, syllabic segmentation; PWPh, production of words from a given phoneme; PhS, phonemic synthesis; PhA, phonemic analysis; IPh, identification of the initial phoneme; WM, phonological working memory; RAN, rapid naming speed using pictures; SR, silent reading; RWNW, reading of words and non-words; AC, auditory comprehension of sentences from pictures.*

Principal Components Analysis (PCA) was carried out to reduce the set of Protocol variables before determining the number of variables that could contribute to the early identification of children with dyslexia, such as skills that are predictors of reading acquisition (**Table 3**); Rotation Method used: Varimax with Kaiser Normalization. All factor loadings greater than or equal to 1.00 were used for interpretation.

**Table 3:** Principal component analysis with extraction sums of squared loadings and rotation sums of squared loadings (Varimax).

	Initial eigenvalues			Extraction sums of squared loadings			Rotation sums of squared loadings		
			
Factor	Total	% of Variance	Cumulative %	Total	% of Variance	Cumulative %	Total	% of Variance	Cumulative %
1	4.233	32.564	32.564	4.233	32.564	32.564	3.024	23.259	23.259
2	2.089	16.069	48.632	2.089	16.069	48.632	2.023	15.565	38.824
3	1.047	8.053	56.685	1.047	8.053	56.685	1.888	14.526	53.350
4	1.010	7.770	64.455	1.010	7.770	64.455	1.444	11.105	64.455
5	0.857	6.592	71.047						
6	0.746	5.736	76.782						
7	0.606	4.665	81.447						
8	0.571	4.394	85.841						
9	0.485	3.734	89.575						
10	0.427	3.288	92.863						
11	0.368	2.827	95.690						
12	0.321	2.472	98.162						
13	0.239	1.838	100.000						


Although 13 components were retained (factors), only 4 accounted for 64.45% of the total variance (eigenvalues > 1). There was a slight change in all variables due to varimax rotation. Analyzing each factor individually, it was found that the first factor explained 32.56% of variance with no rotation with no rotation and 23.25% with rotation. The second factor explained 16.06% with no rotation and 15.55% with rotation. The third factor explained 8.05% with no rotation and 14.52% with rotation, and the fourth factor explained 7.77% with no rotation and 11.10% with rotation. In order to clearly define the groups of variables, a correlation matrix was created employing varimax rotation (a more conservative approach) (**Table 4**).

**Table 4:** Rotated factor loadings in PCA for the four components.

Variable	Pre-reading	Decoding	Reading	Auditory processing
LN (/23)	**0.769^∗^**	0.177	0.134	-0.226
RP (20)	**0.769^∗^**	0.000	0.060	0.254
RI (/20)	**0.756^∗^**	0.076	0.172	0.239
SS (/21)	0.101	-0.064	**0.815^∗^**	0.259
PWPh (/21)	**0.722^∗^**	0.261	0.128	0.002
PhS (/21)	0.268	**0.515^∗^**	0.014	0.408
PhA (/21)	0.067	**0.856^∗^**	-0.005	0.140
IPh (/21)	**0.687^∗^**	-0.123	-0.012	0.444
WM (/24)	0.367	0.192	0.447	**0.470^∗^**
RAN (seconds)	-0.125	**-0.564^∗^**	-0.452	0.181
SR (/10)	0.172	0.339	**0.703^∗^**	-0.177
RWNW (/40)	0.018	**0.657^∗^**	0.507	-0.100
AC (/20)	0.092	0.027	0.003	**0.724^∗^**


*LN, letter-naming; RP, rhyme production; RI, rhyme identification; SS, syllabic segmentation; PWPh, production of words from a given phoneme; PhS, phonemic synthesis; PhA, phonemic analysis; IPh, identification of the initial phoneme; WM, phonological working memory; RAN, rapid naming speed using pictures; SR, silent reading; RWNW, reading of words and non-words; AC, auditory comprehension of sentences from picture. ^∗^*p* < 0.05.*

The first factor, called “pre-reading” had high loadings for five variables, indicating 23.26% of variance for all 13 Protocol variables. The variables Letter-naming (*M* = 21.66/*SD* = 2.88) and Rhyme production (*M* = 11.42/*SD* = 6.96) had the same loading factor, followed by the variables Rhyme identification, Production of words from a given phoneme, and Identification of the initial phoneme. Letter-naming is one of the most important findings referred to as the foundation of other skills in the first school years, when additional skills are developed. This variable has been proved to be influenced by family environment and pre-school literacy instruction. It can be observed the standard deviation of this variable was low for all students. Moreover, Letter-naming has been associated with phonological awareness because most letter names contain clues regarding their corresponding sound. Rhyme Production (*M* = 11.42/*SD* = 6.96) and Rhyme identification (*M* = 15.45/*SD* = 5.09) allow students to realize that words can share identical sound segments, as the perception of greater amounts of sounds will facilitate the formation and increase of lexical and semantic memories, which will be accessed to retrieve auditory information and reading comprehension, afterward. Studies have pointed out that the acquisition of rhymes can occur before literacy instruction, around 3 years old, and it can be combined with skills related to the identification of the initial phonemes, contributing to foster phonemic awareness. Thus, phonemic awareness can emerge as the perception of the smaller segments of spoken words (phonemes), allowing children to perform tasks such as production of words from a given phoneme (*M* = 14.23/*SD* = 8.25) and identify of the initial phoneme (*M* = 15.45/*SD* = 5.09), as well to start establishing phoneme-grapheme correspondence, which is important for reading acquisition. The larger standard deviation of these tests suggests that some of the students evaluated may have had difficulties in accessing a word or a phoneme.

The second factor was called “decoding” since it refers to the ability of using grapheme–phoneme correspondences required to read words. It had four variables with 15.56% of variance. The variable Phonemic analysis had the highest factor loadings, followed by the loadings of positive sign of the variables Phonemic Synthesis and Reading words and non-words and the loading of negative sign of the variable Rapid Naming Speed using pictures, which had negative correlation with the second component. In the RAN test, the naming time is measured and the score is expressed in seconds. Lower scores indicate better performance on the test. The ability to identify phonemes contributes to alphabet comprehension since a phoneme may be represented by a sequence of letters. However, it is important to highlight that the Brazilian Teaching Method does not emphasize teaching letter-sound correspondence. Therefore, it can be said that with regard to the Phonemic Analysis (*M* = 2.76/*SD* = 5.66) and Phonemic Synthesis (*M* = 2.72/*SD* = 4.84), the students had low performance, suggesting that these variables are important predictors for early identification of dyslexia for this Protocol. These performance difficulties also influenced the students’ performance in the Naming Speed Task (*M* = 43.44/*SD* = 10.63) since slow processing speed indicates that the student had difficulties in combining visual with phonological information, suggesting difficulties in reading words and non-words (*M* = 17.15/*SD* = 16.08).

The third factor, called “Reading” had only two variables accounting for 14.56% of variance. Syllabic segmentation (*M* = 19.38/*SD* = 3.41) and Silent reading (*M* = 8.95/*SD* = 1.52) had higher factor loadings. Results indicated that the students had good performance in the Syllabic segmentation test, which does not depend on explicit instruction, and even preschoolers or illiterates have these skills. Silent reading test was the other variable correlated with this factor, which is a specific task that compares recognition between two words and that can be performed by readers without explicit syllable decodification, and the syllables may act as perceptual units in word recognition because of their phonological and orthographic properties, as mentioned by Ashby (2016). Finally, the fourth factor was called “Auditory processing” and comprised the two last variables, accounting for 11.10% of variance and 64.45% of cumulative variance. Auditory Comprehension of sentences from picture (*M* = 19.32/*SD* = 2.51) and the Phonological Working memory (*M* = 20.54/*SD* = 3.05) comprise the last factor. These two variables are somehow correlated because the first one requires that previous information be temporarily stored in the phonological memory (phonological input store until all syntactic and semantic analyses have been completed). **Table 5** shows the distribution of factors, according to the risk criteria – performance below the 25th percentile and at least 51% of the variables correlated with the factors represented by total variance explained by each factor. It can be seen from **Table 5** that 34 students were identified by the first factor, 87 students by the second factor, 19 by the third factor, and 16 by the fourth factor.

**Table 5:** Distribution of the factors according to the risk criteria (performance below the 25th percentile).

Factors	Tests	*n*	*n* (risk criteria <25%)	Minimum	Maximum	*M*	*SD*
Pre-reading	LN (/23)	149	36	5	23	21.66	2.88
	RP (20)	149	38	0	20	11.42	6.96
	RI (/20)	149	35	0	20	15.45	5.09
	PWPh (/21)	149	44	0	22	18.68	3.53
	IPh (/21)	149	43	0	21	14.23	8.25
	Total		34				
Decoding	PhS (/21)	149	108	0	21	2.76	5.66
	PhA (/21)	149	84	0	20	2.72	4.84
	RAN (seconds)	149	145	20	80	43.44	10.63
	RWNW (/40)	149	75	0	40	17.15	16.08
	Total		87				
Reading	SR (/10)	149	39	2	10	8.95	1.52
	SS (/21)	149	43	0	21	19.38	3.41
	Total		19				
Auditory processing	WM (/24)	149	36	1	24	20.54	3.05
	AC (/20)	149	35	0	28	19.32	2.51
	Total		16				


*LN, letter-naming; RP, rhyme production; RI, rhyme identification; SS, syllabic segmentation; PWPh, production of words from a given phoneme; PhS, phonemic synthesis; PhA, phonemic analysis; IPh, identification of the initial phoneme; WM, phonological working memory; RAN, rapid naming speed using pictures; SR, silent reading; RWNW, reading of words and non-words; AC, auditory comprehension of sentences from picture.*

## Discussion

This research presented two studies. In study 1, the results indicated that it was possible to develop a screening protocol for early identification of children at risk for dyslexia in first-grade students, using Brazilian Portuguese stimuli. As for Study 2, Principal Component Analysis revealed four factors accounting for 64.45% variance in all Protocol variables. These factors are consistent with those reported in the National and International literature, and they have been associated with early signs of dyslexia.

Learning how to read in alphabetic systems require the acquisition and domain of associates each distinctive element of visual symbols onto units of sound (phonology). This correspondence process is called phonological recoding (Share, 1995). Nevertheless, this mapping process is influenced by inconsistency in the symbol-to-sound mapping of orthographies. For example, in some Languages it’s possible to notice that one letter or letter cluster can be associated with several sound pronunciations (e.g., English, Danish), whereas in other Languages, such as Italian and Spanish, there is a one-to-one correspondence (one letter per sound). However, in some Languages, such as Portuguese and French, it is possible to find both irregularities and regularities, affecting recoding accuracy, which is in line with the reduced consistency of these languages (Ziegler and Goswami, 2006).

The first factor found was denominated “pre-reading” because its variables can be observed before formal education. The “pre-reading” factor comprised the following tests: Letter-naming, Rhyme Production, Rhyme identification, Production of words from a given phoneme, and Identification of the initial phoneme. Letter naming has been considered as a major indicator because its possibility the association between a letter and sound (letter-to-speech sound integration), which can be impaired in children with dyslexia. Although letter naming is considered to be one of the most important predictors of succeeding reading acquisition. However, it’s important to note that it is strongly influenced by others factors, such as verbal abilities, teaching methods, and parental input. Letter naming is also closely correlated with phonological awareness (Lerner and Lonigan, 2016). The performance of letter knowledge and phonological awareness at kindergarten have been strongly referred as predictors for First-grade reading achievement. These findings were pointed even when variables, such as parental education level and teacher-rated academic competence (Ortiz et al., 2012; Lerner and Lonigan, 2016); Lerner and Lonigan (2016) also discussed the influence of phonological awareness on the acquisition of letter knowledge.

Unfortunately, even though international researchers have pointed out the role of Letter-naming and teaching of letter-naming correspondence in several alphabetic languages, according to the *Parâmetros curriculares nacionais da Língua Portuguesa* (Brasil, 1997) (National curricular parameters of Portuguese Language), the current understanding of the relationship between writing acquisition and writing skills confront entrenched beliefs that the phonics instruction domain is a prerequisite for language teaching, indicating that the two learning processes (literacy and language teaching itself) could occur simultaneously. Therefore, with regard to the Alphabetic language principles, the acquisition of alphabetic knowledge does not guarantee that the student will be able to understand or produce texts in writing. Finally, according to these parameters, teaching basic units could comprise not only reading comprehension, which does not mean that teaching words or sentences would not focus on specific didactic situations that would benefit students.

Perhaps, because of this lack of systematic approach to teaching, letter-naming skills have still been considered as one of the predictors to dyslexia, as reported in international studies on alphabetic language. Kim et al. (2010) and Lerner and Lonigan (2016) argued that for letter names that incorporated important traces of the corresponding sound. Therefore, knowing the name of a letter was a strong predictor to realize if the children knows the corresponding sound. However, this can be observed in children with good developed phonological awareness skills.

According to Lonigan et al. (2009), phonological awareness develops along a *continuum* awareness of large and concrete sound units (i.e., words, syllables) to awareness of small and abstract sound units (i.e., phonemes). The other variables correlated with Factor 1 concern the perception of large amounts of sounds (Rhymes) and the use of phoneme knowledge. The findings of this study showed that students had more difficulties with Identification of the Initial Phoneme than with Production of words from a given phoneme. Therefore, in these phonemic tests, students have to identify the first phoneme of the words (i.e., alliteration) and retrieve another word from the phonological long-term memory. Our results corroborate with those found in the literature suggesting that even before entering preschool, children learn some basic language skills and notions (detection, rhyming, and alliteration) that will facilitate the development of reading skills based on a variety of life experiences. These experiences contribute to their acquisition of receptive vocabulary phonological skills, and narrative understanding and production (Hayes and Slater, 2008; Manz et al., 2010). With regard to Phonemic awareness, as mentioned by Silvén et al. (2002), this finding may support the assumption that conscious access to speech patterns is influenced, at least indirectly, by advances in implicit phonetic and phonotactic representations that can be related to language development during the 1st year of life. Ouellette and Haley (2013) stated that the principal motivation for considering the role of vocabulary in the emergence of phonemic awareness could be associated with the first words stored in mental lexicon. As new words are added, segmental representation becomes necessary so that similar sounding items are not confused with each other. Essentially, increased extensiveness of oral vocabulary causes restructuring, by which there are more specific phonemic-level representations. Accordingly, Law et al. (2016) evaluated a group of pre-reading children with a family risk for dyslexia. As results, the authors founded that there was an influence of phonological and morphological awareness on reading development. According to Morris et al. (2012), morphological awareness can be defined as the explicit awareness and ability to manipulate and reflect upon the morphemic structure of words, which has already been demonstrated in pre-reading children. The results obtained suggest that phonological awareness is a relevant component of morphological awareness, independent of reading experience. It is also important to highlight that in the present study, the variables correlated with factor 2, corroborate those reported in the study of Hulme et al. (2015), who found that children at risk for dyslexia show general deficits in oral language skills in the preschool years. Those deficits are presented in a way that a percentage of these children satisfy the criteria for language impairment diagnosis. Poor oral language skills, in turn, appear to affect the later development of decoding (through problems in acquiring letter-sound knowledge and phoneme awareness) as well as reading comprehension abilities. Based on these international studies, it can be said that the variables correlated with the first factor proved important as predictive variables in the Brazilian Portuguese Language. As an alphabetic language, difficulties in acquiring letter naming and initial phonological awareness skills can be seen as a sign of reading difficulties.

Thus, the 2nd factor was comprised the following tests: Phonemic Synthesis, Phonemic analysis, Rapid Naming Speed using pictures, and Reading of words and non-words. As described by Ouellette and Haley (2013), phonemic awareness can also be categorized based on how it is being used. Specifically, explicit awareness at the level of the phoneme includes both analytic (ability to break a word down into constituent sounds) and synthetic skills (combining sounds together to make a larger segment, such as word). Analysis tasks are more difficult than synthesis tasks. Our findings showed that phonemic analysis and phonemic synthesis had different loadings; however, the students had similar performance (mean) on the tests of phonemic analysis and phonemic synthesis. Furthermore, our findings also showed difficulties in reading words and non-words and a negative loading for Rapid Naming Speed (RAN). This suggests that difficulties in decoding skills were related with slow phonological access. Evaluating the double deficit hypothesis, Brazilian and International studies (Wolf and Bowers, 1999; Andrade et al., 2013; Silva and Capellini, 2013; De Groot et al., 2017), demonstrated the relationship among phonological awareness difficulties, dyslexia, and impaired RAN. Hulme et al. (2015) argued that phoneme awareness and letter knowledge are the most important predictors of early word-reading skills across several languages, and there is evidence of reciprocal interaction between them. Extending these ideas mentioned before (Shaywitz and Shaywitz, 2005; Kirby et al., 2010; Snowling, 2013; Hulme et al., 2015). Kirby et al. (2010) examined RAN effects across languages and the impact of its relationship to reading. These authors also reviewed the instructional literature aiming to improve and to use RAN as a predictor of RTI. They concluded that RAN is uniquely associated with a variety of reading tasks across orthographies, and that the use of RAN measures would be very useful for early identification.

The third factor was called “reading” and had only two variables. It was composed of the tests Syllabic segmentation and Silent reading. Syllabic segmentation is one of the skills that does not depend on explicit instruction. Our results might suggest that students had good performance on Syllabic segmentation, but they had some difficulties in the reading tests. One possible explanation is that in the test of silent reading they could use the visual or orthographic routes instead of the phonological route. Thus, the orthographic process occurs when groups of letters or entire words are processed as single units rather and not as a sequence of grapheme–phoneme correspondences, which is related to phonological processing (Ehri, 1997; Pinheiro et al., 2008; Oliveira and Capellini, 2010; Mota et al., 2012; Majerus and Cowan, 2016). Moreover, Kirby et al. (2010) stated that because of the orthographic process, it’s possible to establish the mechanism of quickly recognition of very frequent or familiar (Morris et al., 1998; Vaessen et al., 2009). Since the reading test was composed of reading words and non-words, both variables correlated with factor 3 may demonstrate that simpler phonological awareness skills (e.g., Syllabic segmentation) can contribute to early identification of dyslexia because syllabic segmentation does not depend on reading instruction and is not related to oral language acquisition. The other variable, the variable related to Reading, will also contribute for early identification of dyslexia since it’s enables evaluation of the reading level, considering the use of the lexical route and the phonological route. In a study addressing cross-language reading comparison, Ziegler and Goswami (2006) reported that one of the most significant findings was that the students who were acquiring reading in orthographically consistent languages (Greek, Finnish, German, Italian, and Spanish) were close to ceiling in both word and non-word reading by the middle of first grade. Unfortunately, this was not observed in the Brazilian population studied. In contrast, the standard deviation of this test for Brazilian Portuguese Language was large (students who were not able to read a single word). According to Scliar-Cabral (2003), Brazilian Portuguese is also considered to be transparent, and reading can be performed only when the grapheme-phoneme matching rules are known and phonological decoding is used, mostly at the beginning of literacy. However, it is worth highlighting that despite the existence of the National curricular parameters of Portuguese language (Brasil, 1997) there is no systematic teaching of grapheme–phoneme correspondence rules. The findings shown in **Table 5** can be justified by the lack of systematic teaching, and because of this, factor 2 (“Decoding”) identified a larger number of students, when compared with factors 1, 3, and 4. Factor 2 was composed of the Phonemic tests Rapid Naming Speed using pictures and Reading of words and non-words.

The 4th factor comprised the tests of Auditory Comprehension of sentences from pictures and Phonological Working memory. A deficit in verbal short-term memory is well documented in dyslexia and can be observed in tasks such as reading longer pseudowords or sequences are used, specially repeating 4 to 6-syllable pseudowords that are related with phonological deficits (Ramus and Ahissar, 2012). Baddeley (1986) defined phonological memory as the coding of information, a sound-based representation system for temporary storage, that can be measured by immediate recall of verbally presented material (e.g., repetition of non-words), as also reported by Lonigan et al. (2009). Studies (Ehri, 1997; Pinheiro et al., 2008; Oliveira and Capellini, 2010; Mota et al., 2012; Majerus and Cowan, 2016) reported that dyslexia children have difficulties in phonological awareness, and this difficulties can still be observed in adults with a history of dyslexia. This difficulty plays an important rule to characterize the dyslexia profile, suggesting that the reduction of the amount of phonological and graphemic information that can be co-activated during the reading process can influence the recoding reading process, when grapheme–phoneme correspondence are not yet automatized, leading to difficulties in reading comprehension. Therefore, it’s important that the children have an efficient phonological memory that enable the maintenance of an accurate representation of the correspondence grapheme–phonemes while word decoding and, consequently, allocate more cognitive resources to comprehension processes. (Lonigan et al., 2009). Spoken-language comprehension and processing depend on the accurate isolation and interpretation of meaningful units of speech such as words, sentences, or utterances. Such high-level perceptual units correspond to the consolidation of basal acoustic-phonetic cues that can be categorized within various time scales corresponding to various phonological grain size units. Therefore, in order to have a good performance in Auditory Comprehension of sentences from pictures, the students evaluated were able to decode phonological information.

Finally, it is possible to identify the variables for the Screening Protocol for Early Identification of Reading Problems in Brazilian children enrolled in first grade, according to the order of the predictive value of each variable, which was as follows: Letter-naming, Rhyme Production, Rhyme identification, Production of words from a given phoneme, Identification of the initial phoneme, Phonemic analysis, Reading of words and non-words, Phonemic Synthesis, Rapid Naming Speed using pictures, Syllabic segmentation, Silent reading, Auditory Comprehension of sentences from picture, and Phonological Working memory. Combined, these three factors (Pre-reading, Decoding, and Reading) accounted for 53.35% of the students’ performance on the Protocol, and thus these factors would be statically sufficient to create a version of the protocol proposed. However, future studies are necessary to verify the exclusion of the 4th factor (Auditory processing).

Our findings are in agreement with those found by Thompson et al. (2015), who indicated that early identification of “reading problems” is difficult, and the development of assessment protocols for this age and grade level are extremely important, as they can prevent future learning damages. Furthermore, our findings also suggest that early language problems can be considered as risk factors for dyslexia, but they can be also considered as risk factors for this disability for children entering school. Although Principal components Analysis revealed four factors, it is important to highlight that future analysis are still necessary to investigate the underlying factors affecting test items. However, if we take into account the educational reality in Brazil, the screening protocol proposed accomplished one of its main goals, which is helping professionals, such as teachers, Speech Language Therapists, and others to identify students at risk for dyslexia or other reading problems in 1st grade, since this type of protocols are practically non-existent in the country. In Brazil, one of the issues related to the identification of children at risk for dyslexia is the long period of time until they are referred to diagnostic centers. This results is consistent with the view that many children who have language delay and receive proper treatment can learn how to read. However, it is worth mentioning that these children continue at risk of having difficulties in reading skills and can present others difficulties, including social problems.

## Conclusion

Results indicated that screening protocol developed in the present study showed four major factors: pre-reading (Letter-naming, Rhyme production, Rhyme identification, Production of words from a given phoneme, Identification of the initial phoneme); decodification (Phonemic synthesis, Phonemic analysis, Rapid Naming Speed using pictures, Reading of words and non-words); reading (Silent Reading and Syllabic segmentation); and Auditory processing (Phonological working memory and comprehension of sentences from pictures) to identify Brazilian Portuguese speaking children at risk for dyslexia.

Based on the PCA carried out, our findings showed the effective use of the proposed Screening Protocol to analyze the predictive factors that can explain later reading achievement.

## Ethics Statement

This study was carried out in accordance with the recommendations of ‘Frontiers guidelines’ with written informed consent from all subjects. All subjects gave written informed consent in accordance with the Declaration of Helsinki. The protocol was approved by the ‘Ethics Committee of Faculty of Philosophy and Sciences, São Paulo State University “Júlio de Mesquita Filho” (FFC/UNESP).’

## Author Contributions

GG, AC, and SC had substantial contributions to the conception or design of the work; or the acquisition, analysis, or interpretation of data for the work; drafting the work or revising it critically for important intellectual content; final approval of the version to be published; agreement to be accountable for all aspects of the work in ensuring that questions related to the accuracy or integrity of any part of the work are appropriately investigated and resolved.

## Conflict of Interest Statement

The authors declare that the research was conducted in the absence of any commercial or financial relationships that could be construed as a potential conflict of interest.
